# Decreasing the Epilepsy Treatment Gap in Tena, Ecuador: A Report From 2021 to 2023

**DOI:** 10.7759/cureus.94581

**Published:** 2025-10-14

**Authors:** Grace Bayas, Gema Bayas, Angel Bayas, Erica O Bayas Cardenas, Noralyn Franco, Christian M Sigcha, Kevin Shapiro, Shaye Moskowitz, Patricio S Espinosa, Rajib Kanti Dey, Chester S Camia, Steve Coates, Charlotte Stow, Niels Turley, Jin Jing, Michael B Westover

**Affiliations:** 1 Neurology, Beth Israel Deaconess Medical Center, Harvard Medical School, Boston, USA; 2 Neurology, International Neurology Foundation, Boca Raton, USA; 3 Neurology, Espinosa Neuroscience Institute, Boca Raton, USA; 4 Interventional Radiology, Boca Raton Regional Hospital, Boca Raton, USA; 5 Medical School, Florida Atlantic University, Boca Raton, USA; 6 Medical School, Universidad de Las Américas, Quito, ECU; 7 Neurology, Children’s Hospital Los Angeles, Los Angeles, USA; 8 Neurosurgery, Broward Health, Pompano, USA; 9 Neurology, Boca Raton Regional Hospital, Boca Raton, USA; 10 Neurodiagnostics, Cortica, Los Angeles, USA; 11 Neurophysiology, TeleEEG, Whitley Bay, GBR; 12 Neurology, TeleEEG, Barnard Castle, GBR

**Keywords:** diagnostic gap, ecuador, epilepsy care, global health, health equity, neurological care, neurology, rural health, telemedicine, underserved populations

## Abstract

Background

Neurological care in rural areas such as Tena, Ecuador, remains critically low due to geographic, economic, and systemic barriers. Tena, located in the Amazon region, has limited access to specialized neurological services, creating significant health disparities. Since 2009, the International Neurology Foundation (INF) has partnered with the Hospital José María Velasco Ibarra to address these challenges.

Methodology

This retrospective analysis summarizes data from the INF medical service relief trip (MSRT) conducted in Tena from 2021 to 2023. Clinical records, interviews with providers, and MSRT reports were reviewed to assess patient demographics, diagnoses, treatments, and interventions. Descriptive statistics and thematic analysis were used to identify trends and insights.

Results

Over three years, 751 patients were treated, with epilepsy being the most common diagnosis (265 cases). Children under the age of 10 years represented the largest patient group. Key achievements included the donation of electroencephalography equipment, enabling local epilepsy diagnostics, and training local healthcare providers. Persistent challenges included limited imaging resources, inconsistent medication supply, and barriers related to language and transportation.

Conclusions

INF’s initiatives have significantly improved access to neurological care in Tena, enhancing diagnostic capabilities and providing critical training. Sustainable progress requires investment in infrastructure, expanded training programs, and consistent medication availability. The Tena experience serves as a model for reducing health disparities and improving neurological care in resource-limited settings, aligning with global health equity priorities.

## Introduction

In the heart of the Ecuadorian Amazon, along the Napo River, lies Tena, a town of approximately 80,816 inhabitants [1]. Tena serves as the capital of Napo province and one of the most populous cities in the Amazon region, known locally as El Oriente (the East). Despite its significant population, the region has long suffered from a critical lack of access to specialized healthcare, particularly in neurology. The Medical Specialist Distribution in Ecuador Study by Rodriguez et al. found that in 2017, Ecuador had only 220 neurologists for a population of over 17 million. The provinces of Pichincha and Azuay had a higher rate of specialists compared to other provinces, and the Amazon region was shown to have the fewest specialists [2]. Similar to the study by Rodriguez et al., Moreno-Zambrano et al. also found concerning gaps in Ecuador’s neurology workforce, reporting that by 2023, only 94 neurologists remained in the country, with a national ratio of just 0.54 per 100,000 population, and only one neurology training program located in Quito [3].

Neurological conditions, including epilepsy and stroke, represent a substantial global burden of disease, particularly in low- and middle-income countries, where access to care is often limited by geographic, economic, and systemic barriers [4,5]. Stroke, for example, is the second leading cause of death worldwide and requires urgent, specialized care to reduce mortality and disability [4,6]. Recent studies have shown that neurological disorders disproportionately affect racial and ethnic minorities, individuals from lower socioeconomic backgrounds, and those living in underserved regions, including rural areas and low-income countries [4,7]. Tena exemplifies these challenges, facing significant gaps in access to specialized neurological care and diagnostic services. These barriers are further compounded by linguistic differences, as many indigenous communities primarily speak Quechua and Shuar, leading to communication challenges between patients and physicians, in addition to widespread economic hardship and limited transportation infrastructure [8,9]. These local challenges reflect broader global health priorities, including the World Health Organization’s emphasis on achieving universal health coverage and ensuring equitable access to essential services [10,11].

In response to these challenges, the International Neurology Foundation (INF) has partnered with the Ministry of Public Health Hospital José María Velasco Ibarra (HJMVI) to address the neurological care gap in Tena. Since 2009, the INF has conducted annual medical service relief trips (MSRTs), deploying multidisciplinary teams of adult and pediatric neurologists, neurosurgeons, nurse practitioners, and volunteers to provide care [12]. While these efforts have brought temporary relief, they also underscore the need for sustainable solutions, including investments in local infrastructure, training programs, and medication availability.

This report summarizes the INF’s work over a three-year period (2021-2023), highlighting patient demographics, the burden of neurological disease, and the resources available in Tena. By sharing these insights, we aim to inspire other neurologists to contribute to similar initiatives and advocate for systemic changes that would allow for more sustainable and equitable neurology care in Tena and other underserved regions. Ultimately, this effort aligns with broader global goals to reduce healthcare disparities and improve outcomes for communities in resource-limited settings.

## Materials and methods

Study design and setting

This report is a retrospective, descriptive analysis of the neurological care provided in Tena, Ecuador, during the INF annual MSRTs from 2021 to 2023. The neurological care consists of annual follow-up checkups, electroencephalography (EEG) recordings if necessary, and referrals to specialists if necessary. These MSRTs were conducted at the Ministry of Public Health HJMVI, the primary public healthcare facility in the region. The data presented here reflect the MSRTs’ clinical activities, patient demographics, diagnoses, and interventions during this period.

Data collection

Clinical Records

Patient data, including demographics, diagnoses, and treatments, were extracted from medical records maintained during the MSRTs. These records included electronic and paper charts completed by the INF team with the support of local healthcare providers.

Interviews With Providers

Insights into challenges, resource limitations, and the evolving capacity of local healthcare infrastructure were gathered through semi-structured discussions with INF neurologists, local physicians, and hospital administrators. Participants were selected based on their direct involvement in the MSRTs.

MSRT Reports and Documentation

Annual reports and operational documentation from the INF MSRTs were reviewed to compile information on resources donated, training sessions conducted, and specific interventions.

Data analysis

Descriptive statistics were used to summarize patient demographics, diagnoses, and treatment patterns over the three-year period. Trends were identified through year-over-year comparisons of key metrics, such as the number of patients treated and the types of conditions diagnosed. Qualitative insights from provider interviews were analyzed thematically to identify recurring challenges and opportunities for improvement.

Ethical considerations

This study was reviewed by the Beth Israel Deaconess Medical Center Committee on Clinical Investigations (protocol number: 2025P000216) and was determined to be exempt from institutional review board protocol requirements under Exempt Category 4, in accordance with federal regulations for retrospective studies. This determination was made on April 14, 2025, and formally communicated on May 14, 2025. A HIPAA Waiver of Authorization was also granted. All patient data were fully de-identified to protect privacy, and insights gathered from provider discussions were anonymized to ensure confidentiality. As this report evaluates programmatic implementation rather than individual clinical outcomes, it falls within the scope of exempt research.

## Results

Demographics

The Amazon region of Ecuador, including Tena, is home to a diverse population, with a significant proportion identifying as Indigenous, primarily from the Quechua and Shuar communities. Nationally, Indigenous peoples make up approximately 7.7% of Ecuador’s population, with higher concentrations in the Amazon. According to linguistic and demographic studies, Spanish is spoken as a first language by over 90% of Ecuadorians and as a first or second language by more than 98%, but 2% of the national population speaks only an Indigenous language  [13,14]. Quechua and Shuar are constitutionally recognized as official intercultural languages, yet in practice, formal healthcare, including at HJMVI, is typically delivered in Spanish. This is a language gap that can hinder communication of diagnosis, understanding of treatment plans, and adherence to medications [13-15].

Economic disparities further exacerbate healthcare access challenges. Many residents of the Amazon rely on subsistence agriculture, with limited financial resources to seek care. High poverty rates in the region restrict access to both preventive and specialized healthcare. Additionally, geographical barriers complicate travel to HJMVI, as many communities are in remote areas accessible only by unpaved roads or rivers. For these residents, reaching the hospital can require up to four hours of travel by car and up to five hours via public transportation, at considerable cost. Along with land transportation, numerous patients travel via canoe from the depths of the Amazon to be cared for by our team in Tena, Ecuador. The canoe mode of transportation can take up to four hours from their respective homes. As a result, many patients turn to local clinics, which frequently lack the resources to diagnose or treat neurological conditions adequately. Traditional healers, shamans, also play a central role in healthcare delivery in these communities, offering both spiritual and physical care. The integration of these practices with Western medicine presents unique challenges and opportunities for collaboration [15].

From 2021 to 2023, the INF MSRTs served a growing number of patients, reflecting an increasing demand for neurological care in Tena. Across the three years, a total of 719 patients were treated. The majority were children under the age of 10 years, who comprised 167 of the 331 patients seen in 2023. This pattern suggests a probable high prevalence of developmental and neurological conditions among children in the region, though further research is needed to confirm this. The distribution of male and female patients varied slightly by year; in 2023, for example, 171 male and 160 female patients were treated. Patient demographic data, including age and sex, were extracted from MSRT records. Ethnicity data were not recorded; however, as the MSRTs were conducted exclusively in Tena, Ecuador, the study population predominantly consisted of local native Ecuadorian residents.

Understanding the demographic, cultural, and socioeconomic context of Tena is critical for tailoring healthcare interventions. Addressing these barriers, by providing linguistically and culturally appropriate care, improving transportation options, and ensuring economic feasibility, can enhance the effectiveness of MSRTs and promote better health outcomes for the region’s underserved populations.

Neurology resources in Tena

Neurology resources in Tena are extremely limited. HJMVI, where the INF MSRT is based, operates without neurologists, except during the annual three-day MSRT. Patients in need of neurological evaluations must travel to one of the larger cities in the central region of Ecuador, most often Quito or Ambato. Traveling to these cities from Tena takes approximately four hours by public transportation. Many patients who travel from deeper Amazon regions can take up to seven hours, having to travel by canoe, foot, and then public transportation to reach these cities. Basic neurological diagnostics are generally either unavailable or unreliable, as simply getting to the hospital in those regions makes the trip too tedious for many patients to follow through with it.

Before 2021, EEG for the diagnosis of seizures and epilepsy was not available in Tena except during the three days of the MSRT. In 2021, the INF, in collaboration with TeleEEG, donated equipment for EEG to HJMVI, significantly enhancing the hospital’s diagnostic capabilities. TeleEEG is a UK-based nonprofit organization that provides EEG equipment, training, and remote interpretation services to under-resourced regions around the world [16]. Selected hospital staff also received intensive training in the technical aspects of EEG recording. After recording, the studies are uploaded to a cloud server and interpreted remotely by an INF neurologist. However, because there are no local EEG technicians with significant experience, EEG recordings are of variable quality.

Imaging services are also limited. A CT scanner, essential for diagnosing a range of neurological conditions, including congenital anomalies, neoplasms, and acquired brain injuries, was operational in 2023 but was unavailable in 2021 and 2022 due to maintenance issues and a lack of technical staff. When the CT scanner in Tena is not operational, patients must be transported to the nearest regional hospital in Puyo, approximately 90 minutes away. MRI is not available in the Amazon region through the public health system, and patients requiring MRI must either pay out of pocket for private radiology services or travel several hours, at their own expense, to a public hospital with this technology. Patients requiring neurosurgical procedures, such as those with hydrocephalus needing shunt placement, must be transferred to Quito. Some patients suffer irreversible neurological damage before they can access specialized care.

HJMVI provides a limited range of drugs, including some anti-seizure medications, free of charge through the Ministry of Public Health. Some other medications are available for purchase in local pharmacies. However, the restricted availability of neurological medications hampers effective long-term management of conditions such as epilepsy.

Table 1 provides the data on medications prescribed by the INF MSRTs from 2021 to 2023. Medications are categorized based on the primary diagnoses treated, including epilepsy, psychiatric disorders, migraines, and Parkinson’s disease.

**Table 1:** Medications prescribed by the INF MSRTs to Tena, Ecuador, in 2021, 2022, and 2023. INF = International Neurology Foundation; MSRT = medical service relief trip

	2021	2022	2023
Epilepsy			
Valproic acid (mg and mg/mL)	40	54	69
Carbamazepine (mg)	25	27	37
Clonazepam (mg)	3	6	11
Lamotrigine (mg)	10	7	10
Levetiracetam (mg)	5	4	5
Clobazam (mg)	2	0	2
Gabapentin (mg)	0	1	1
Phenobarbital (mg)	2	0	1
Phenytoin (mg)	1	0	1
Depakote (mg)	0	0	1
Pregabalin (mg)	1	1	0
Psychiatric			
Amitriptyline (mg)	4	9	17
Risperidone (mg)	6	1	7
Methylphenidate (mg)	0	0	7
Guanfacine (mg)	0	0	3
Fluoxetine (mg)	1	0	2
Sertraline (mg)	0	1	2
Quetiapine (mg)	2	0	2
Mirtazapine (mg)	0	0	1
Alprazolam (mg)	0	0	1
Aripiprazole (mg)	0	0	1
Compazine (mg)	0	0	1
Haloperidol (mg)	2	1	1
Ritalin (mg)	0	0	1
Escitalopram (mg)	2	1	0
Citalopram (mg)	1	0	0
Migraine			
Acetaminophen (mg)	4	1	10
Riboflavin (mg)	0	0	8
Risperidone (mg)	0	0	7
Propranolol (mg)	0	0	3
Ibuprofen (mg)	1	3	2
Aspirin (mg)	0	0	1
Migradoxin (mg)	2	0	1
Migrex (mg)	0	0	1
Naproxen (mg)	1	0	0
Parkinson’s disease			
Carbidopa levodopa (mg)	5	8	5
Biperiden (mg)	0	0	1
Mirapex (mg)	0	1	0

Until a few years ago, medical records in Tena consisted entirely of paper charts. Over the past five years, HJMVI has moved toward electronic medical records, although paper charts are still in use as well. Responsibility for maintaining records and imaging from outside the hospital, however, falls on patients, who must bring documentation and imaging studies (on media or film) to appointments.

Neurology care provided by the INF MSRTs

Since 2009, the INF has mobilized a variety of neurologic professionals as well as residents, students, and other volunteers from the United States and Ecuador to provide care in Tena. In Tena, the INF team works alongside local pediatricians, medical students, and residents, who participate in the MSRTs. As shown in Table 2, the INF MSRTs treated over 700 patients between 2021 and 2023, with the majority being children under the age of 10 years.

**Table 2:** Age and sex distrubution of patients seen by INF MSRTs in Tena, Ecuador, in 2021, 2022, and 2023. INF = International Neurology Foundation; MSRT = medical service relief trip

2021			
Age group	Male	Female	Total
0–10	37	42	79
11–17	18	18	36
18–59	23	26	49
60+	7	9	16
Total	85	95	180
2022			
Age group	Male	Female	Total
0–10	66	40	106
11–17	18	26	44
18–59	33	36	69
60+	13	8	21
Total	130	110	240
2023			
Age group	Male	Female	Total
0–10	97	70	167
11–17	33	31	64
18–59	33	39	72
60+	8	20	28
Total	171	160	331

The data presented in Table 2 outlines the demographics of patients seen by the INF MSRTs over the course of three years (2021, 2022, and 2023). Patient age groups were divided into the following four categories: 0-10, 11-17, 18-59, and the advanced age of 60+. In 2021, a total of 180 patients were seen, with slightly more females (95) than males (85). Patient turnout in 2021 was slightly lower relative to later years, perhaps as an indirect result of the COVID-19 pandemic, including local lockdowns, transportation barriers, and reduced hospital accessibility during that period. By 2022, the total increased to 240, with males (130) slightly outnumbering females (110). The 0-10-year age group had the highest number of patients across all three years, peaking in 2023 with 167 children. In 2023, the total number of patients reached 331, with males (171) again slightly outnumbering females (160). Every year, the number of patients seen continues to grow. The increase in patient attendance observed in 2023 likely reflects both the easing of COVID-19-related restrictions and greater community awareness, which is typically spread through word of mouth among local residents.

Epilepsy was the most commonly diagnosed condition, with 265 cases recorded over the three-year period, as shown in Table 3. This was followed by developmental and learning disabilities (90 cases) and migraines (75 cases). Other common conditions included speech disabilities (55) and autism (61). Conditions such as traumatic brain injury, cerebral palsy, and Parkinson’s disease were less common, with fewer than 40 cases over the three-year span. Overall, 719 diagnoses were made, with a sharp increase in patient visits and diagnoses in 2023.

**Table 3:** Diagnosed conditions seen by the INF MSRTs in Tena, Ecuador, in 2021, 2022, and 2023. INF = International Neurology Foundation; MSRT = medical service relief trip

	2021	2022	2023	Total
Epilepsy	73	80	112	265
Development and learning disability	20	37	33	90
Migraine	28	14	36	75
Speech disability	18	8	29	55
Traumatic brain injury	10	15	12	37
Autism	8	10	43	61
Cerebral palsy	8	11	11	30
Parkinson’s disease	8	12	4	24
Microcephaly	1	7	8	16
Down syndrome	1	7	6	14
Macrocephaly	2	3	7	12
Other	13	10	17	40
Total	197	204	318	719

In the analysis presented in Table 3, among the diagnosed conditions, epilepsy was categorized as a single group. Seizure subtype information (e.g., focal vs. generalized epilepsy) was not consistently specified in the medical charts and was therefore excluded from the analysis.

Epilepsy remained the most common diagnosis encountered. Of the 147 EEGs performed over three years (50 in 2021, 51 in 2022, and 46 in 2023), a wide range of EEG features were identified, as summarized in Table 4.

**Table 4:** Yearly distribution of EEG features recorded during INF MSRTs (2021–2023). INF = International Neurology Foundation; MSRT = medical service relief trip; EEG = electroencephalography

EEG feature	2021	2022	2023
Generalized theta slowing	1	0	4
Generalized delta slowing	0	0	2
Posterior dominant rhythm	0	0	27
Generalized polyspikes	0	0	1
Φ sleep	0	0	4
Diffuse theta slowing	1	4	3
Diffuse delta slowing	0	0	7
Interictal epileptiform discharges	1	0	3
Generalized spike-and-wave	3	7	3
Vertex waves	0	0	10
K-complexes	0	0	6
Sleep spindles	0	0	5
Non-REM stage 1 sleep	0	0	5
Non-REM stage 2 sleep	0	0	8
Epileptiform discharges	3	6	3
Focal delta slowing	3	3	1
Irregular delta slowing	0	0	1
Sharp waves	2	0	1
Alpha rhythm	0	0	1

The EEG feature data for 2021 and 2022 represent only the findings documented in the INF’s medical records. It is unclear whether cases without recorded abnormalities reflected normal EEGs or were left unreported; thus, only recorded findings were included in this analysis. The 2023 data are more comprehensive, reflecting improved consistency in EEG documentation. Seizure subtype data were available only for a limited number of patients and were therefore excluded from the analysis due to inconsistent documentation. Anti-epileptic drug treatment is often initiated or modified during the MSRTs. Most in-person follow-ups occur during the next annual MSRT. Patients remain vulnerable to interruptions in medication due to inconsistent supply and, in many cases, poor medical literacy, which can reduce treatment adherence.

## Discussion

Challenges and opportunities

Despite significant progress, several systemic challenges persist in addressing the neurological care gap in Tena. While the INF MSRTs have made strides in introducing EEG technology, training healthcare providers, and improving medication access, sustainable solutions require further investment in infrastructure, workforce development, and community engagement.

Diagnostic resources, including imaging capabilities, remain insufficient. Although the operational CT scanner at HJMVI has improved diagnostic capacity, frequent maintenance issues and staffing shortages hinder consistent use. The lack of MRI technology in the Amazon region forces patients to travel significant distances or incur prohibitive costs, delaying critical care. Expanding access to reliable imaging is essential to reducing diagnostic delays and improving outcomes.

Capacity building is another priority. While the INF MSRTs have conducted training sessions on epilepsy and stroke management, establishing continuous professional development programs for local healthcare providers is crucial. Telemedicine platforms such as TeleEEG offer an opportunity to provide year-round support, remote consultations, and ongoing education, ensuring sustained improvements in care delivery [16].

Medication access remains a critical barrier. Although some anti-epileptic drugs are available through the public healthcare system, gaps in supply disrupt treatment continuity. Advocacy to expand the formulary and establish reliable supply chains can address these gaps, improving adherence and long-term outcomes.

Addressing social determinants of health is vital. Economic hardships, linguistic diversity, and transportation barriers significantly impact access to care. Strategies such as subsidizing transportation costs, creating linguistically appropriate educational materials, and collaborating with traditional healers could enhance accessibility and foster trust within the community [15].

The unique sociocultural dynamics of the region must be considered. Indigenous communities often seek traditional healers, and language barriers frequently obstruct care delivery. Collaborating with traditional healthcare structures and creating linguistically appropriate materials can enhance accessibility and trust [15].

While these challenges reflect broader systemic gaps in neurological care across resource-limited regions, the INF MSRTs demonstrate that scalable, collaborative solutions are possible [12]. By investing in infrastructure, training, and telemedicine, Tena can serve as a model for sustainable, equitable healthcare delivery.

Accomplishments of the INF MSRTs

The INF MSRTs have made a tangible impact on neurological care in Tena, with notable accomplishments in patient outcomes, local capacity building, and resource provision. Over the past three years, the MSRTs have treated more than 700 patients, most of whom had limited or no prior access to specialized neurology care. Epilepsy, the most common diagnosis, has been managed successfully in many patients, with several remaining seizure-free for years due to tailored treatment plans and ongoing follow-up.

One key achievement is the integration of EEG services into the local healthcare system through the donation of equipment by TeleEEG in 2021. Since then, 147 EEGs have been performed, significantly improving the diagnosis and management of epilepsy. These efforts have enabled earlier interventions and reduced delays in care, particularly for children, who comprise a substantial proportion of the patient population. While challenges with EEG quality remain, the availability of this technology marks a major milestone in improving diagnostic capabilities in the region.

The INF MSRTs’ educational initiatives have also strengthened local capacity. Through workshops and lectures, the MSRTs have trained healthcare providers on critical topics such as status epilepticus, epilepsy management, and stroke care. This training has enhanced the ability of local practitioners to manage neurological emergencies, ensuring that care can continue between annual MSRTs. Telemedicine platforms such as TeleEEG provide remote support and education, and their potential to enable sustainable growth in neurologic care is clear [16]. For example, local pediatricians and general practitioners now routinely use protocols introduced during the MSRTs to stabilize and manage epilepsy cases.

Patient success stories illustrate the MSRTs’ broader impact. For instance, in one typical case, a child was diagnosed with epilepsy during the 2021 MSRT and started on a tailored treatment plan. This child has remained seizure-free for over two years, allowing her to return to school and participate fully in daily life. Such outcomes demonstrate the transformative potential of providing specialized care in underserved settings.

The INF MSRTs’ model of combining patient care, education, and advocacy underscores its commitment to creating long-term change. Sustained investment in infrastructure, supply chains, and professional development will be essential to achieve long-term impact. While there is still significant work to be done, these accomplishments provide a foundation for building a more sustainable and self-sufficient neurological care system in Tena and beyond. This model can serve as a blueprint for reducing neurological disparities in other resource-limited settings.

Limitations

This report reflects observations made during a short-term MSRT, which limits the ability to assess long-term outcomes or systemic changes. Due to constraints in time and resources, we were not able to collect comprehensive quantitative data, such as treatment outcomes or diagnostic timelines, which would provide a more complete picture of impact. Additionally, input from patients, local providers, and health administrators was not systematically gathered, which may limit the depth of insight into community perspectives. Because data were retrospectively collected from MSRTs’ records, there is potential for selection bias and incomplete documentation, particularly in EEG findings. Reliance on retrospective reports may also introduce recall or reporting bias. Lastly, as the challenges and strategies discussed are specific to the context of Tena, the findings may not be generalizable to other regions with different healthcare infrastructures or sociopolitical dynamics.

## Conclusions

The urgent need for neurological care in Tena underscores the broader challenges of healthcare delivery in resource-limited regions. The INF MSRTs have made meaningful progress through a multifaceted approach, including the introduction of EEG technology, targeted training for healthcare providers, and advocacy for improved medication access. These efforts have enhanced diagnostic accuracy, improved epilepsy management, and laid the foundation for sustainable care. The Tena experience highlights the transformative potential of collaborative, community-driven initiatives. Scaling this model will require continued investment in infrastructure, particularly imaging capabilities, and the development of continuous education programs supported by telemedicine platforms such as TeleEEG. The experience in Tena highlights the impact of collaborative, community-centered solutions and offers a promising blueprint for expanding equitable neurological care in underserved regions around the world.
